# Advocacy and Open Science in the UK: Case Studies in the Autism Wars

**DOI:** 10.1007/s40617-023-00881-2

**Published:** 2023-11-28

**Authors:** Mickey Keenan, Karola Dillenburger

**Affiliations:** 1 School of Psychology, Ulster University, Cromore Road, Coleraine, BT52, 1SA Northern Ireland UK; 2 Queen’s University Belfast, Belfast, Northern Ireland, UK

**Keywords:** Advocacy, Autism, UK, Practice guidelines, Applied behavior analysis, ABA, British Psychological Society, BPS, Open science, Fake news

## Abstract

Individuals on the autism spectrum experience a wide range of support needs and it comes as no surprise that opinions differ as to the best way to provide necessary supports. Some articulate self-advocates argue that societal acceptance of neurodiversity is the key issue. These views have clashed with those of parents and professionals who advocate for access to evidence-based interventions for profoundly autistic children and adults. The consequences of these kinds of differing opinions are so far-reaching that the term “autism wars” was coined. In this article, we argue that although acceptance of diversity is obviously important, this should include an openness to diverse scientific traditions, especially if lack of such openness limits public policy and adversely affects individuals and families. “Open Science” holds much promise in many fields, but its influence cannot be taken for granted when it comes to evidence-based support practices that are grounded in the science of behavior analysis. Benefiting from open science in autism research requires well-developed advocacy skills. To illustrate, we use case studies from the UK, where advocates of open science have met with intractable obstacles.

During the late 2010s, a significant anti-science movement seemed to dominate much of public discourse (Portes, 2017). Terms such as “fake news” and “post-truth” became common parlance in discussions about economics, climate, health, and education (Lazer et al., 2018; Molina et al., 2019; Greifeneder et al., 2020; Tandoc et al., 2017), often via social media, to describe the dissemination of false or misleading information (Wardle & Derakhshan, 2017) or to denote “circumstances in which objective facts are ***less*** influential in shaping public opinion than what appeals to emotion and personal belief” (Oxford Dictionary, 2016).

The COVID-19 pandemic heralded a shift back to the recognition of the importance of hard facts and good science and it also brought into sharp focus the need for change in the way that research is conducted (Besançon et al., 2021). Research used to be conducted behind a veil of secrecy with findings communicated via an arduous process of peer review and patenting, before eventually being published in journals that were behind a paywall, available only to subscribers. COVID-19 brought with it a significant shift to a different paradigm: open science.

On a most basic level, open science is defined as “the idea that scientific knowledge of all kinds should be openly shared as early as is practical in the discovery process” (Nielson, 2011). Burke and Lees (2023) writing in the British Psychological Society’s (BPS’s) newsletter *(The Psychologist)* about the replication crisis in psychology, note that “Widespread adoption of open science methodologies has been proposed as a solution to improve the robustness of research findings.”

Right at the start of the pandemic, scientists agreed to engage in open science with regards to researching the origins and properties of, as well as mitigations against, the new virus. This led to successful international collaborations that made significant strides in sequencing the virus and developing vaccines at a pace that was previously unthinkable. Michael M. Crow, the president of Arizona State University and Greg Tananbaum, the director of the Open Research Funders Group, summed up the advantages of the move to open science:

Two clear conclusions can be drawn from this rapid alignment. First, the daily workings of science have practical ramifications in all our lives. Scientific norms affect not just researchers working in labs, but also policy makers, doctors, patients, families, and the general public. Second, open science is the form of research dissemination and global collaboration that best reduces vexing limits to knowledge that are exacerbated by COVID-19. If rapidly and openly sharing research data and papers is critical to understanding and combating coronavirus, doesn’t the same hold true for cancer? Heart disease? Climate change? The scientific community—moving with great speed and clarity of purpose—has clearly signalled that open science is the most efficient way to tackle issues that have a significant and direct effect on the lives of the general public. The unambiguous conclusion is that open is better for science. (Crow & Tananbaum, 2020)

Although open science may not be the panacea against all problems with science communication (Kappel & Holmen, 2019; Lupia, 2013) and may not be supported universally by all scientists (Knoche, 2020; Koerber, 2021), its use throughout the COVID-19 pandemic had the potential to reestablish public confidence in the scientific process (Hong & Moran, 2019). Of course, shifts in public perception are difficult to achieve and even more difficult to quantify. Doubt about science generally will remain for some people, as is evident in skepticism about COVID vaccines. Climate change denial is another example where open science is not embraced (Byrne, 2020). Nonetheless, Dye et al. (2021) conclude “clear and careful scientific communication is paramount in relaying support for scientific-based policies.”

With regards to psychology (Open Science Collaboration, 2015), the American Psychological Association supports open science as a vital “set of practices that increase the transparency and accessibility of scientific research” (Hong & Moran, 2019). It is clear that open science and interdisciplinary communication have the potential to influence policy decisions more directly and more rapidly than more traditional “academic silo” approaches (Paepcke-Hjeltness, 2021).

The growing support for open science indirectly calls out those who continue to work behind closed doors and who by their actions or omissions evidence an ostensible bias against other sciences or scientists (Watson, 2015). This is particularly important when their actions have a negative impact on public policy, with the result that support is withheld from those who need it, such as profoundly autistic people^1^ (Lord et al., 2021). In this case, promotion of open science is synonymous with advocacy for autistic individuals who need supports. This is not the first time that problems with closed minds, omission, and misinformation about ABA have been pointed out either by us (Dillenburger et al., 2014; Dillenburger, Jordan et al., 2015a, Dillenburger, Keenan et al., 2015b; Dillenburger & Keenan, 2009, 2023; Keenan et al., 2015; Keenan & Dillenburger, 2018b, 2020b) or others (Gorycki et al., 2020; Leaf et al., 2018, 2022; Morris, 2009). It seems that Brandolini (2013) may have been right when he observed that “The amount of energy needed to refute b . . . s . . t is an order of magnitude bigger than that needed to produce it” (quote adjusted by authors). It is in this spirit that we try again and in this article we describe some specific examples that document problems arising from the failure to embrace open science practices, particularly in terms of misinforming public policy makers.

## Autism Guidelines Development

The prevalence of autism diagnosis is rapidly growing, with 2%–4% of children and at least 1% of adults now considered to be on the spectrum (Centers for Disease Control & Prevention [CDC], 2023; Dillenburger, Jordan et al., 2015a, Dillenburger, Keenan et al., 2015b). Although autism is considered a medical diagnosis (American Psychiatric Association [APA], 2013), there are no medical tests, and diagnosticians rely purely on behavioral observations. There are two main behavioral diagnostic categories/criteria: (1) persistent social communication and interaction issues and (2) at least two different persistent restricted, repetitive patterns of behavior, interests, activities (including “sensory issues”). According to diagnostic manuals, individuals with a diagnosis of autism are assessed as having either some (Level 1), substantial (Level 2), or very substantial (Level 3) support needs (Disability Scoop, 2022).

Guidelines on how best to support autistic children and adults are developed by teams of professionals who conduct systematic reviews of existing research evidence. Comprehensive systematic reviews generally conclude that nearly all evidence-based autism interventions are based on the applications of knowledge accumulated by the science of behavior analysis (e.g., Myers & Johnson, 2007; National Autism Center [NAC], 2009, 2015; Surgeon General, 1999; see also Cambridge Centre for Behavioral Studies, 2023). Reviews that focus exclusively on group design research evidence the effectiveness of behavioral interventions (Dixon et al., 2021; Eikeseth et al., 2002; Smith, 2012; Smith & Iadarola, 2015; Smith et al., 2007), but at times come to more guarded conclusions (Howlin et al., 2009; Magiati et al., 2012; National Institute of Health Care & Excellence [NICE], 2013; Rodgers et al., 2021).

Behavior analysis is a natural science that investigates fundamental principles of behavior and its applied branch (Applied Behavior Analysis [ABA]) uses these principles to support and facilitate the development of behavior that is socially important^2^ (Chiesa, 2020; Cooper et al., 2020). The mainstay research methodologies used in behavior analysis are single-system research designs (SSRD; Johnston & Pennypacker, 2008; What Works Clearing House [WWC], 2010, 2022), where the behavior of the participant prior to the intervention serves as a control for behavior observed during and after the intervention (Hanley, 2017). For reasons outlined in significant detail elsewhere (Green, 2010; Johnston & Pennypacker, 2008; Keenan & Dillenburger, 2011) and in line with the evolving area of precision medicine (Ginsburg & Phillips, 2018), traditional methodologies, such as randomized controlled trails (RCT) or other group average-based designs generally are considered not suitably sensitive to assess individualized behavior analytic interventions and indeed are inappropriate for assessing any scientific discipline (Keenan & Dillenburger, 2011). Yet, in the past SSRDs have been excluded from many systematic reviews of autism research (Grigorenko et al., 2018; Kratochwill & Levin, 2014; NICE, 2013), that consequently omitted important findings of evidence-based interventions. In recent research, SSRDs have been recognized as a valid and important research methodologies (WWC, 2010), that should not be ignored by future reviews of research regarding evidence-based interventions for children and youth on the autism spectrum.

More and more, findings from behavior analysis are shared in the spirit of open science, via open access books (Dillenburger, 2015), websites (Association of Professional Behavior Analysts [APBA], 2017; Cambridge Centre for Behavioral Studies, 2020; Centre for Behaviour Analysis [CBA], 2010; Keenan & Dillenburger, 2020a; Kennedy Krieger Institute, 2018; Princeton Child Development Institute, 2023), and social media (O’Donnell, 2021; Simons Powering Autism Research, 2023; The Behavioral Observations Podcast, 2023; Keenan & Dillenburger, 2019, 2023a), where research data, animated illustrations, podcasts, and recordings of high-caliber webinars and lectures are shared freely for anyone to peruse (Gilroy & Kaplan, 2019; Unumb, 2013; Keenan & Dillenburger, 2023b). By contrast in the UK, there are instances that do not reflect the spirit of inclusiveness and open science and that consequently affect negatively on public policy developments. To illustrate the importance of advocacy in these cases, we share here our experiences with these kinds of situations.

The first example is the BPS approach in the production of their guidelines for autism interventions.

The British Psychological Society is the representative body for psychology and psychologists in the United Kingdom and the only body in the UK which covers all areas of psychology. Broadly, the Society aims to raise standards of training and practice in psychology, raise public awareness of psychology, and increase the influence of psychology practice in society. (NAS, 2023)

In terms of influencing UK policy, the BPS say: “Our work, and the work of our members, helps to influence and develop a psychological approach to policy-making. . .” (BPS, 2023). Elsewhere, they note:

More broadly, the Policy Unit is engaged in a wide range of activities across the parliaments and assemblies in the UK to increase the opportunities for the input of psychological expertise in policy development. Through both the APPG [All Party Parliamentary Group] and our broader outreach, we have dramatically extended our network of key Parliamentarians and are regularly providing briefings, parliamentary questions and suggested debate or Early Day Motion topics. (BPS, 2018)

Given the extent of the societal reach of the BPS, it behooves them to ensure that information they bring to policy makers is entirely accurate. In 2006, the BPS published its first autism guidelines. The document was brief and to the point (BPS, 2006). In 2014, in light of emerging evidence about autism, the BPS set up a small working group to revise these guidelines. The review group included representatives from three of the UK countries, including an autistic adult. The initial drafts of the new guidelines underwent multiple in-depth and cross-divisional consultations and revisions. The new guidelines were reviewed by the BPS Policy Team before finally, in 2016, being signed-off by the BPS Practice Board, published on the BPS website, and distributed widely on BPS social media (BPS, 2016). The new 17-page guidelines included the following statement related to the use of clinical services based on the science of behavior analysis:

In the UK, psychological treatment for ASD has traditionally been offered by a psychologist, however, behavior analysis-based interventions should be supervised and/or delivered by Board Certified Behavior Analysts (BCBA). Most BCBAs have a background in Psychology and it is noted that a growing number are part of/lead multidisciplinary autism teams. Note that this document does not recommend that BCBAs should supplant psychologists, but recognises their contribution to the supervision and/or delivery of interventions, depending upon the specific needs of the individual client. (BPS, 2016, p. 4)

This statement was based on evidence accrued over 50 years of research in behavioral health care in support of individuals on the autism spectrum that showed, as outlined above, that best practice is based on principles of ABA. There are hundreds if not thousands of peer-reviewed published studies, often using SSDR and many impartial independent systematic reviews that confirm these findings (Larsson, 2021). As a result of this evidence, the Surgeon General (1999) confirmed that ABA-based interventions were to be at the heart of mainstream community care for individuals with autism in the United States. A similar approach was taken in Canada (Autism Society Canada, 2004) and elsewhere (Kelly et al., 2018). The paragraph in the 2016 BPS guidelines simply stated that these kinds of interventions should be supervised by suitably qualified professionals.

Approximately 1 week after the publication of the 2016 BPS autism guidelines, the BPS withdrew the document, apparently without informing either the public, the chairperson, or any other member of the review group. These professionals had given of their time and expertise for 2 years, free of charge, and, via multiple reviews and revisions, had ensured that the document was socially valid and inclusive of the voice of people on the autism spectrum. A reason for the withdrawal was not given. This had the potential not only to malign the professional integrity of the authors of the report, but also to undermine the credibility of the BPS in the eyes of stakeholders and policy makers.

As a consequence of these events, the chair of the 2016 BPS autism guidelines review group resigned, not only as chair of the review group, but also from his over 20-year membership of the BPS. Moreover, in publicity distributed later, the entire 2016 BPS guidelines development work appears to have fallen prey to the vicissitudes of *damnatio memoriae* (BPS, 2021d).

After intense protestations from parents who were advocating for their autistic children and who had welcomed the 2016 BPS autism guidelines, the BPS convened a “consensus” meeting in London in early December 2016. The invitees consisted of 16 psychologists, 3 of whom were certified behavior analysts (Behavior Analyst Certification Board [BACB], 2023) whereas a considerable number of the other attendees were on record for their contentious views about behavior analytic autism interventions (references to individuals are withheld to ensure confidentiality). There was no-one in the meeting to represent the autistic voice (Benevides et al., 2020) or those with profound autism (Lord et al., 2021).

Because the London meeting was held under the Chatham House Rules (Chatham House, 2021), all we can say is that the discussion was heated and there was no consensus at the end of the meeting. In fact, at one point, one of the attendees even suggested that the 2016 BPS autism guidelines were an attempted *coup d’état* by behavior analysts.

The psychologists present at the London meeting are not the only psychologists with controversial views about behavior analysis (Hughes, 2008; Jordan, 2001; The Skeptical Advisor, 2014). For example, Baron-Cohen (2014), from the Autism Research Center at Cambridge University, whose theories have been heavily criticized by autistic adults (e.g., Boon, 2022), controversially suggested that behaviorism, the philosophy that underpins behavior analysis, is dead and of historical interest only. One could ask where such hostility comes from given that the scientific evidence in favor of behavior analytic supports and interventions to support individuals on the autism spectrum is so overwhelming especially when compared to other interventions (Dillenburger, 2015; Howard et al., 2014; New York State Department of Health, 2011; Warren et al., 2011).

It seems that one of the key factors is the lack of good quality third-level educational opportunities in behavior analysis in the UK and elsewhere, when compared to the United States (Association for Behavior Analysis-International [ABAI], 2023). Well-trained students of behavior analysis understand the scientific concepts and methods that underpin behavior analysis. Todd and Morris (1983) pointed out that a major source of the hostility towards behavior analysis lies in inaccurate coverage in college textbooks that are used in the initial training of psychologists and policy makers:

To the extent that public policy is shaped by individuals whose exposure to behaviorism is through textbooks . . . and through educators who assign those textbooks, then these policies and decisions are not likely to reflect the important conceptual and applied contributions that a natural science of behavior can offer. (p. 158)

When fallacious or incomplete information is published in general psychology textbooks (Morris, 2009), it is not surprising that omissions and inaccurate information about behavior analysis find their way into reports (NICE, 2013) and newsletters (Howlin, 2013). The problems are compounded further when arguments based on restricted access to apposite information are taken up by autistic adults, who then allege that behavior analysts work to a normalization agenda that is based on ableist views (Milton, 2012). Ableism is the belief that disability is deviant and represents an unwanted difference (Tarvainen, 2019). A noncircular definition of normalization (i.e., that does not include the word “normal”) could not be found; for example, in the *Oxford English Dictionary*, generally considered the world’s most authoritative sources on the English language, normalizing is defined as “bring or return to a *normal* or standard condition or state” (emphasis added). In behavior analysis, this translates into the provision of services for people with disabilities such that they can avail of the benefits associated with regular circumstances and ways of living.

Neither ableism nor normalizing are endorsed within the science of behavior analysis. In fact, it has long been acknowledged that key to the discipline of behavior analysis is its focus on the analysis of environmental contingencies and how these affect behavior, thus aiming to enhance understanding and acceptance of individual differences (Cooper et al., 2020; Moore, 2013; Walsh, 1997; Wolf, 1978) and the human spectrum of diversity (Dillenburger & Keenan, 2023; Murray et al., 2023). Without question, there are historical reports that early intensive behavior intervention resulted in such significant skills development that some children become “indistinguishable” from their neurotypical peers or that they presented with “normal educational or intellectual functioning” (Lovaas, 1987). Yet, the historical use of terms that are less in line with today’s conceptualization of autism does not mean that behavior analysts valued these children differently from other children, or that the intended goal of intervention was to normalize these children (Baer, 2004; Dillenburger & Keenan, 2023; Wolf, 1978).

In ABA-based programs, target behavior and intervention procedures are individually tailored in collaboration with the person themselves or their caregivers (in the case of a very young child). Each child is valued and validated as a unique person in their own right through individualized supports (Fein et al., 2013). As a result, in general outcomes are aimed at empowering each individual with enhanced or new skills that are relevant to their personal circumstances and that affect their quality of life (Mayer et al., 2011).

Of course, it is important that the views of autistic people are taken into account (Leaf et al., 2022; Schuck et al., 2022); they are significant partners in the debate about autism (Milton, 2018; Murray et al., 2023). However, an autistic self-advocate who may require some adjustments to be able to carry out their job at a university or in a computer firm (e.g., Level 1) is very different from someone who requires significant support with very basic life- and selfcare-skills or significant self-injurious behavior (e.g., Level 3). If the distinction in levels of support needs is not fully embraced (APA, 2013), the discussion is incomplete (Schuck et al., 2022; Singer et al., 2022).

Although behavior analysts can provide important services to autistic persons with needs at Level 1, who are seeking support for issues such as social interactions, self-management, and anger management, for the most part in the UK, behavior analysts are employed to assist people who have significant (e.g., Level 2) or very significant (e.g., Level 3) support needs (Brodhead et al., 2018; Chiesa, 2006; NICE, 2012). As a result, the people who are going to miss out when open science is not embraced in the field of autism are those most severely affected. In fact, the National Council on Severe Autism in the United States, who represents those who cannot represent themselves, advocates for a distinct diagnosis of profound autism (cf., Lord et al., 2021). Maybe such a distinction would alleviate the sometimes acrimonious debate between self-advocates and parent advocates (Demchak et al., 2020; Garner et al., 2022); it might ensure that progress is not stymied and energies are not wasted on inaccuracies that are antithetical to the principles of open science (Brandolini, 2013).

In other words, behavior analysts do not assume that there is one best way of living one’s life (Dillenburger & Keenan, 2023). Behavior analysis defines behavior as the interaction between an organism and their environment with a focus on learning history across the lifespan, present contingencies, and culture (Cooper et al., 2020). As such, although behavior analysts acknowledge the importance of biological factors (Schneider, 2012), the focus of interventions is on the creation of supportive contingencies/environments that lead to social empowerment of service users (Wolf, 1978).

As far as the London “consensus” meeting is concerned, it ended with the BPS’s decision that there would be an open call for additional members for a panel, to work alongside the original authors of the 2016 guidelines to draft yet another new BPS autism guidelines document (see minutes of BPS Autism Consensus Meeting, London, May 12, 2016). Yet, subsequent to the London meeting, the original authors of the 2016 guidelines were informed that they would not be included in the new panel automatically but that they would have to submit a fresh statement of interest for inclusion in the new panel (BPS personal communication, 2017). It eventually transpired, however, that the new 2021 BPS autism guidelines (BPS, 2021c) were published without input from any the 2016 BPS autism review panel members, without comprehensive membership consultation, and seemingly without “experts by experience” (i.e., persons on the autism spectrum; Care Quality Commission, 2021) on the panel (see membership of the Task and Finish Group; BPS, 2021c). These are all actions that arguably raise questions for policy makers about the governance of the professional body that provides the evidence on which they are supposed to base their policies.

## Overview of the Critique of the New BPS guidelines

Regarding the 2021 BPS autism guidelines document, there are some points that are useful and commendable and there are a number of issues that are contentious. For example, there is a good discussion about neurodiversity and autism diagnosis. Furthermore, the guidelines also provide helpful information regarding assessment. On the other hand, it could be argued that some of the language used throughout the document is ableist and guilty of labelling (Benevides et al., 2020); starting with the title “Working with autism” (p. 1). The term “autism” is a diagnostic summary label (Cooper et al., 2020) and in their basic training, psychologists learn that they should focus on people, not diagnostic labels (Keenan et al., 2021). Therefore, most professionals use people-first language (Kenny et al., 2016). At the same time, of course, identity-first language often preferred by autistic adults is equally acceptable (Singer et al., 2022). Thus, much better titles for the 2021 BPS autism guidelines could have been used, such as “Working with people on the autism spectrum” (Bottema-Beutel et al., 2020) or “Working with autistic people” (Benevides et al., 2020; Kenny et al., 2016).

Apart from the issue with ableist language, one could object to the contention that autism necessarily is lifelong (BPS, 2021d, p. 7), when there is statistically significant evidence to the contrary (Fein et al., 2013; Orinstein et al., 2014). This does not mean that there is a “cure” for autism, as autism is not an illness that requires a cure (Orinstein et al., 2014). It simply means that because autism is diagnosed when certain behaviors are observed (Disability Scoop, 2022), if these behaviors change over time then the diagnosis might change. Of course, this principle applies to most psychological diagnoses and is therefore not exclusive to autism.

Readers of the 2021 BPS guidelines could be astounded also at the outdatedness of the prevalence figures cited, which state that 1% of the population is diagnosed with autism (BPS, 2021d, p. 7), when recent figures show much higher prevalence rates, especially for children (2%–4.2%; CDC, 2023; Dillenburger, Jordan et al., 2015a, Dillenburger, Keenan et al., 2015b; Department of Health, 2020; Kim et al., 2011). The document also leaves itself open to critique for being formulated from within a medical model rather than a social model with a strong focus on disorder and disability, despite the fact that there is an acknowledgement that many autistic adults reject these terms. There also is an issue with what could be construed as a normalizing agenda as indicated by a focus on deficits without balancing this with a strength-based approach (Social Care Institute for Excellence, 2021).

Although it is not the purpose of this article to address these issues in depth, it is important for policy makers to be cognizant of factual inaccuracies and cultural insensitivity (i.e., autistic culture, e.g., Dekker, 2013). Here, we focus on evidence of selection bias when portraying research on interventions based on applied behavior analysis. This points towards the need for effective advocacy in cases of blatant misrepresentation of another science. In the 2021 BPS autism guidelines (BPS, 2021d), applied behavior analysis is summarily dismissed in one sentence based on a single reference:

Although intensive ABA^3^ programmes have been found to have positive effects on IQ and adaptive behavior two years after intervention, there is no evidence that they reduce severity of autism or improve longer-term outcomes (Rodgers et al., 2021). (p. 24)

This single sentence stands in stark contrast to hundreds, if not thousands, of national and international peer-reviewed research papers, systematic reviews, and practice guidelines evaluating autism interventions based on applied behavior analysis that have been published over the past 50 years (APBA, 2017; Cambridge Centre for Behavioral Studies, 2023). In addition, the new BPS guidelines ignore the National Institute for Clinical Excellence (NICE) guidelines for the management of children and young people with autism (NICE, 2013) that clearly acknowledge the significance of ABA: “Applied behaviour analysis (ABA) is a general approach to intervention that can involve a wide range of behavioural strategies and can be used to change behaviours across multiple domains” (NICE, 2013, p. 27).

In fact, NICE guideline NG11 recommends that behavior analysts should be on the team when working with individuals whose behavior present challenges to service providers (NICE, 2015, Section 1.1.5). Earlier, NICE had developed separate general public health guidance on behavior change (NICE, 2007) and guidelines on individual approaches (NICE, 2014). Although these earlier documents did not specifically mention ABA, the urgent need for effective theoretical as well as practice approaches to behavior change was clear even then.

The 2021 BPS autism guidelines do not reference any of these NICE guidelines although they reference two other NICE Clinical Guidelines (CG170; NICE, 2013) an update (CG128; NICE, 2011), and CG142 (NICE, 2012). They also do not reference other international endorsements of ABA-based interventions, such as those in all 50 states in the United States, who introduced legislation to ensure that behavior analytic interventions are available for children on the autism spectrum who need them (Autism Speaks, 2018). The new 2021 BPS autism guidelines also ignore the fact that similarly strong endorsements are in place in Canada (Laucius, 2019; Motiwala et al., 2006), Norway (Oien, 2016), and the Netherlands (Peters-Scheffer et al., 2010, 2011, 2012), as well as across a wide range of professional organizations in the United States (Cambridge Centre for Behavioral Studies, 2023).

## The Antithesis of Open Science

Within the 2021 BPS guidelines, the exclusion of any reference to the large body of evidence garnered from the application of the science of behavior analysis is clearly at odds with the principle of open science. The scant mention and immediate dismissal of outcomes from a different scientific tradition in one sentence of a 52-page document, presents an unbalanced set of guidelines. It could indicate that neither the values of open science, nor providing an evidence base were the *raison d’être* that underpinned the production of these guidelines. The question remains as to the rationale behind the incomplete portrayal of applied behavior analysis in the 2021 BPS guidelines. In a different but related context, Graber and Graber (2023) noted that some would prefer to just abolish ABA altogether. If this were in fact the case for the BPS, it would be tantamount to censorship and clearly not in line with open science practices. Crow and Tananbaum (2020) are positive that successful collaboration in open science is not defined by censorship or exclusion but by inclusion:

When knowledge and innovation rest in the hands of the few, we struggle to reach our collective potential. Access to data and published research democratizes information and allows more voices to join the scientific conversation. It removes a layer of insularity in ways both big and small.

The failure of genuine engagement with the available evidence about ABA is also seen where the 2021 BPS autism guidelines recommends two specific intervention approaches: (1) functional analysis (Ayllon & Michael, 1959; Beavers et al., 2013; Lerman et al., 2013) and (2) positive behavior support (PBS; Dunlap et al., 2008; Johnston et al., 2006; Weiss et al., 1992). Both of these intervention approaches originate in (and are applications of) behavior analysis. Their promotion in the 2021 BPS guidelines, while at the same time dismissing their origins only serves to illustrate the need for a critical examination of the influence of outworn shibboleths. In concert with this, there is an urgent need to advocate for accurate representation of research findings.

To be clear, we are not saying that there is nothing to be criticized within the practice of some behavior analysts (Garner et al., 2022; Keenan et al., 2010; Millman, 2020; Schuck et al., 2021), or that behavior analysts have nothing to learn from a neurodiverse analysis (Graber & Graber, 2023), but professional practice and a science are two different things. As in any other profession, the likelihood has to be accepted that some behavior analysts may have engaged in poor practice (Devita-Raeburn, 2016). This may have been the case because of financial pressures, lack of training/acknowledgement of the need for training, and/or poor supervision. There are others who may not have focused sufficiently on client-centered and culturally sensitive socially important behavior that should be the focus of interventions in accordance with the original definition of ABA (Baer, 2004; Baer et al., 1968, 1987; Cooper et al., 2020). As with other professions, in behavior analysis professional regulation and strict ethical guidelines are in place (BACB, 2023; UK Society for Behaviour Analysis (UK-SBA), 2023). Malpractice should be dealt with via relevant professional bodies, national laws, and/or the courts, whichever is appropriate, regardless of a professional title (e.g., Gunn, 2010).

Although it is important to acknowledge shortcomings in some professional practices, it is vital not to throw the baby out with the bathwater. It is not the science itself that is at fault for unprofessional conduct of some (Green, 2016). Given that science is a byword for specific activities of people, it evolves continuously in both cultural and historical contexts. People who act in good faith given the knowledge and training available to them at one time, may indeed need to adjust their behavior when more and new scientific information becomes available or when cultural priorities change. A famous quotation from Maya Angelou captures the essence of this process: “Do the best you can until you know better. Then when you know better, do better.”

## Openness is Better for Science

The importance of openness and collaboration in support for autistic individuals, especially during early childhood, cannot be overestimated. For over 25 years in the UK, many parents of these children have had to engage in advocacy along legal lines via tribunals, where they had to fight for their child’s right to benefit from ABA-based support [the Special Educational Needs (SEN) Tribunals system started in 1994] (Blakemore, 2021; Byrne & Byrne, 2000). In these tribunals, misinformation about behavior analysis has routinely been given as evidence by local authority legal representatives and witnesses, including educational psychologists (Blakemore, 2021; Keenan & Dillenburger, 2020b). At the same time as ridiculing behavior analysts, some actually have admitted to not being an expert in behavior analysis (WalesOnline, 2005). As soon as the 2021 BPS autism guidelines came out, reference to them was added to the catalogue of misinformation used in evidence in tribunals to justify not funding services based on ABA (Blakemore, 2021).

Without good quality behavior analytic services, children on the autism spectrum are much more likely to experience unfavorable long-term outcomes (Howlin et al., 2004), including not progressing in terms of IQ or language, and engaging in behavior that challenges (Howlin et al., 2014). Hollins (2021) lamented the fate of many people diagnosed with autism and called for radical change in autism interventions in the UK: "Autistic people are being locked away in institutions. A radical change is needed.”

The section on psychological interventions in the 2021 BPS autism guidelines states that the BPS does not want to promote specific procedures or interventions; “[t]here is no ‘one size fits all’ approach to autism” (BPS, 2021d, p. 23). In principle, of course, there is nothing wrong with that statement. However, in a context where misinformation about ABA is rampant (Leaf et al., 2018; The Skeptical Advisor, 2014) the ‘one size fits all’ phrase morphs into a ruse against investing in services based on this discipline (Keenan & Dillenburger, 2018a, 2020b). At the heart of the problem is a category mistake in which the discipline is viewed as a specific intervention (Chiesa, 2006), as was the case when the minister for education in Northern Ireland wrote that ABA is “one of many commercially available interventions for children with autism” (Ruane, 2009).

Addressing the questions about progress in autism research, the chair of the 2021 BPS autism guidelines wrote in an earlier article that “. . . although the findings are encouraging there is still no evidence that any one high quality, specific intervention is superior to any other. . .” (Howlin, 2013, p. 5). The article continued, “. . . claims that two years of EIBI [Early Intensive Behavioral Intervention, which is one of the many applied behavior analytic interventions for young children with autism] result in significant lifetime savings are totally spurious” (Howlin, 2013, p. 6). This simply is not an accurate account of the evidence base (NAC, 2015; New York State Department of Health, 2011; Surgeon General, 1999) and there is ample evidence internationally of short- and long-term cost savings for behavior analytic early interventions, such as EIBI (Hume et al., 2020; Motiwala et al., 2006; Peters-Scheffer et al., 2012; Piccininni et al., 2017; Steinbrenner et al., 2020; Wong et al., 2014).

Other historical events suggest that there is something seriously amiss within the BPS generally (BPS, 2021a; Conway & Pilgrim, 2022; Craig et al., 2021) and, in particular, when it comes to their relationship with, or understanding of the discipline of applied behavior analysis and the value of single-system research designs in evidence-based practice. In the early 2000s, a small parent-led charity in N. Ireland asked the BPS for help in providing an impartial review of the Northern Ireland Task Group on Autism report (Department of Education Northern Ireland, 2002). The Task Group report included significant misrepresentations of applied behavior analysis that were negatively affecting service provision for children on the autism spectrum (Parents Education as Autism Therapists, 2023). However, instead of supporting the parents, the BPS was supportive of the damaging views about applied behavior analysis expressed in the Task Group report. Dismayed by the BPS response, the parent advocates sought support from the European Association for Behaviour Analysis (EABA). The EABA invited the BPS to a meeting saying, ”[w]e believe that much could be gained from a reasoned dialogue about the field of behaviour analysis” (EABA, 2008). The BPS declined the meeting stating “. . . we do not view it as appropriate for the Society to be involved in follow-up meetings to its written statements” (BPS, 2008).

**Fig. 1:**
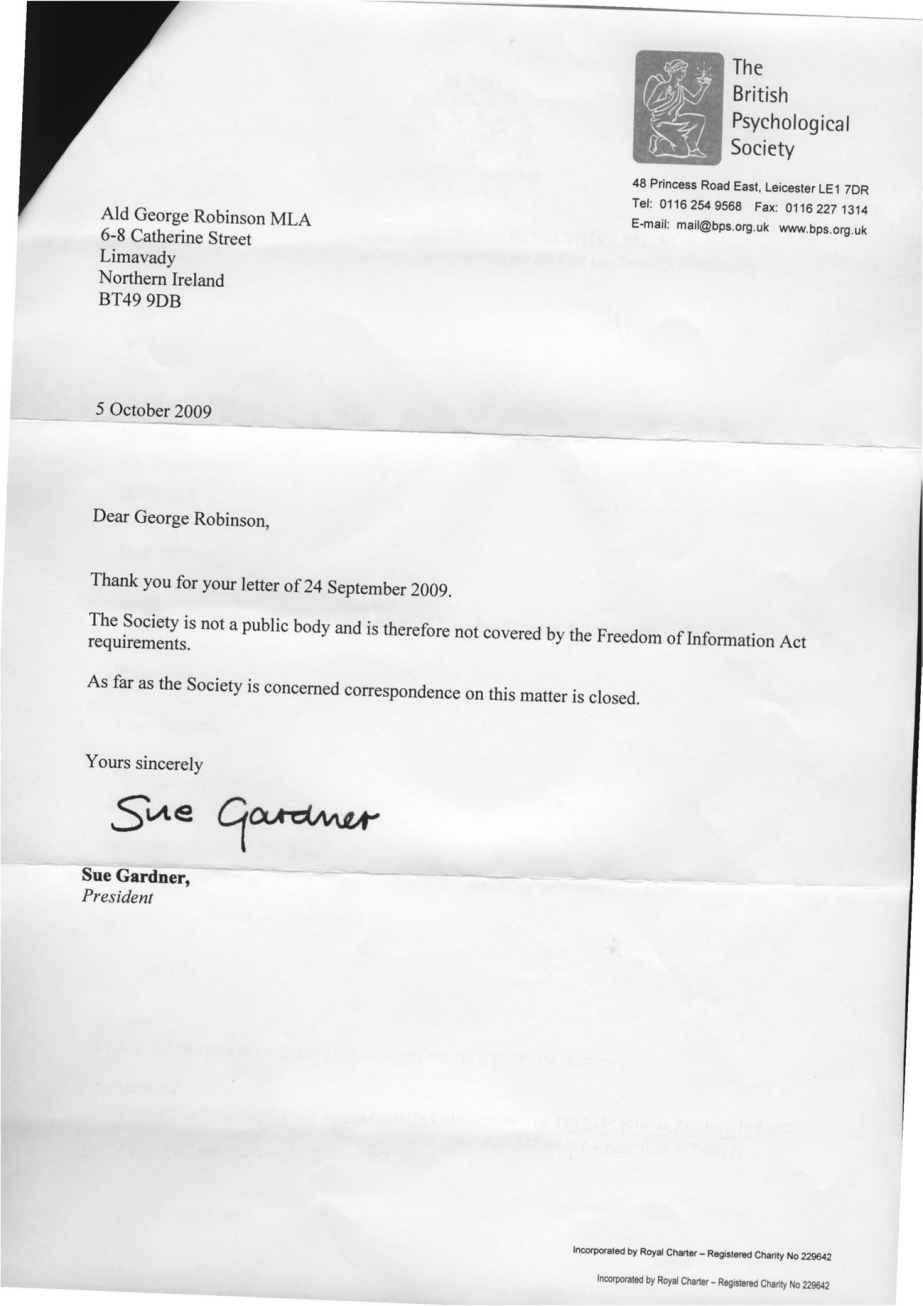
Letter from BPS to Alderman Robinson MLA

Undeterred, the Northern Irish parents turned to a politician for help. Alderman George Robinson, member of Northern Ireland’s Legislative Assembly (MLA), wrote to the BPS asking for information about their view of the Northern Ireland Task Group report: “I am of the understanding that no one with specialist knowledge of ABA was included in the review panel. I therefore have some questions I would appreciate if you could answer for me. . . .” He added a number of questions about panel members and their expertise in applied behavior analysis, and continued, “. . . I am sure you will appreciate that if no one with local knowledge and expertise was involved in the review any results are open to question which serves no one. . .” (Robinson, 2009). The BPS responded by shutting down any further communication: “As far as the Society is concerned correspondence in this matter is closed” (BPS, 2009; Fig. 1). A few years later, the BPS published a special edition on autism in *The Psychologist* (BPS, 2014) without mentioning any of the research findings from ABA-based research.

There are damaging real-life consequences that arise from closed door policies, such as shutting down discussions or not answering legitimate questions (Keenan, 2015; Maginnis, 2008), dismissing concerns of families (Gambrill, 2013), or withdrawing autism guidelines that identified a role for behavior analysts in autism interventions (BPS, 2016). Children on the autism spectrum miss out when misinformation about educational opportunities influences policy and tribunal decisions (Blakemore, 2021) or clinical decision making (Ruane, 2009). In fact, this has gone so far that some behavior analysts feel they have to hide the fact that they are behavior analysts when they try to ensure continuity between ABA-based home programs and schools. Others have been told that “ABA would conflict with existing practices” or “Just don’t say it’s ABA, call it something else” (Parent, 2017).

Many parents of children on the autism spectrum have similar experiences. They feel they have to hide the fact that they know more about behavior analysis than the professionals who are employed to support their children (Dillenburger et al., 2010). In one such case, a parent was told statutory services would be withdrawn if she continued to use these interventions at home (Keenan & Dillenburger, 2020b); in another case, the child was expelled from school when the headmaster discovered that the child was in a home program based on ABA (Parent, 2017). Schools that have obtained excellent inspection results frequently do not disclose the fact that they base their pedagogy on ABA (Smyth et al., 2006).

Parents who put their experiences of ABA on record can play an important role in advocating for ABA to be endorsed in public policy. The account in Table 1 is a good example of their determination not to be silenced; at the same time, it tackles head on the accusation of ableism directed at ABA.

**Table 1:** Description of Problems in Accessing Behaviour Analytic Interventions by a Parent (reprinted with permission Dillenburger, Jordan et al., 2015a, Dillenburger, Keenan et al., 2015b), 12–14)

In a SEN [Special Educational Needs] magazine, a noted behavioural expert Brad Bezilla said that the UK seems to be lagging behind the US when it comes to using [Applied Behaviour Analysis] to manage autistic children’s behaviour. It’s far worse than ‘lagging behind’, Brad. I have stopped counting the number of professionals who have actually tried to put me off using ABA for my autistic boy, even as I tell them how well it works for him. Or who have point blank refused to fund it. Or the horrified looks on the faces of Local Authority (LA) bodies when I mention that I am using ABA. You’d think I’d just confessed to using witchcraft!
It’s very odd. I can only assume that, somewhere along the line, a memo went around to all LA education departments, and especially speech and language therapists (SALTs), saying something like: ‘ABA is the devil’s work – please endeavour to nip any attempts at ABA on the part of deluded parents in the bud very quickly. Tell them that the speech it purports to give their child is not ‘real’ speech, is simply robotic and rote-learned. Tell them that it will ‘disrupt family life‘ (actually, not as much as an aggressive, unmanaged autistic child will, thanks); tell them that it will create too much rigidity in your child, and all that he learns will simply be ‘grafted on’, not properly, intrinsically learned.’
For a while I believed all the above. Until I met a mother who had actually used ABA, and who absolutely refused to let me trot out my LA-inspired nonsense, but told me that her boy had only started to speak after work with ABA tutors, and that his behaviours and stims (self-stimulatory behaviours, e.g., hand flapping) had been immeasurably improved under ABA. Luckily, I went with her, not the LA.
The problem is that the state ‘industry’ around autism is brainwashed into hating ABA, and has only its pet (and wholly unproven) education systems of TEACCH [Treatment and Education of Autistic and related Communications Handicapped Children], and visual timetables to offer. I would like one day to do a survey of the speech and language reports being given out daily to autistic children by SALTs [speech and language therapists] employed by local authorities. I am willing to bet quite a lot of cash that every single report contains the following 4 recommendations – ‘the child needs a low stimulus environment’; ‘the child needs to use Makaton’; ‘the child needs a TEACCH learning approach’; ‘the child needs a visual timetable’. I spent thousands on a much lauded pre-school autistic nursery which specialised in all of the above – my son got worse, not better. Yet, 3 weeks after starting ABA, his speech and his behaviours were 80% improved. And the same has happened for very many of my pals.
The truth is that no-one is measuring how very ineffective our state autism provision actually is – probably on the basis that ‘those little kiddies will never amount to much, it’s not even worth measuring their progress as there’ll never be any’. As a parent, it makes me want to scream. My boy would have been written off - aged 3 - as a no-hoper, had I not found an alternative education for him. I could weep for all those hundreds of autistic 3-year-olds being written off across the UK every day of the week. Kids who will never speak, or read, or write, or live an independent life, because the state has such low expectations of them.
Every single autistic child seems to be getting the same generic recommendations – yet every autistic child is an individual, something that our LAs ought to agree with as it’s enshrined in law in this country. A one-size-fits-all approach, such as is being used with this constant flinging at parents of visual timetables and Makaton, with zero behavioural advice, is a scandal.

These kinds of experiences are common especially among parents of children with profound autism (Blakemore, 2021; Dillenburger et al., 2010; Keenan et al., 2000). In fact, increasingly these parents speak up for their children, advocating for more support (Belmonte, 2021; Coffey, 2021).

Decisions about autism interventions typically are based on professional practice guidelines. This means that the production of best practice guidelines is a serious undertaking because of the clinical as well as policy implications that arise from such a document. Although the 2021 BPS autism guidelines make some useful general points (BPS, 2021c), unfortunately, this new document does not benefit from the democratization of information that characterizes open science (Bezjak et al., 2018). This means that parents and professionals need to advocate for the children who need behavior analytic supports.

There are many professional bodies in behavior analysis (e.g., ABAI, 2023; APBA, 2017; BACB, 2023) that advocate successfully for ABA in the United States to ensure autism guidelines accurately reflect relevant extensive research evidence regarding behavior analysis (Larsson, 2021). In general, ABA lies outside the scope of competency of most psychologists, unless they have undertaken additional training (e.g., MSc ABA; see ABAI, 2023). As a result, when the BPS does not consult with professional bodies in behavior analysis, they have a problem. This problem extends to the BPS *Code of Ethics and Conduct* (BPS, 2021b), which states that “[m]embers should not provide professional services that are outside their areas of knowledge, skill, training and experience” (p. 6). Furthermore, the 2021 BPS panel apparently also did not seem to include autistic adults on the panel (see list of panel members [BPS, 2021c]).

The major achievements in the United States in relation to ABA arose because of the scientific evidence and the unrelenting advocacy and lobbying by parents of children on the autism spectrum, educating and consulting with legislators and regulators, as well as testifying at agency and legislative hearings (Unumb, 2013). Similar developments happened in the Czech Republic (Gandalovičová, 2016) and could happen in the UK as well, but only if the BPS followed the guiding light of open science to remove any outdated vestiges of fake news and disinformation and focused instead on protecting the rights of children (United Nations Convention on the Rights of the Child, 2001) and others who seek support services (United Nations Convention on the Rights of Persons with Disabilities, 2006).

## Implications of Advocacy for Autistic Children

In this article we have discussed the importance of advocacy when it comes to ensuring that autistic children are able to avail of behavior analytic interventions, if and when they need them. We have given a number of practical examples of advocacy in action, such as parents asking questions of professional bodies or challenging erroneous descriptions of behavior analysis and defending their children’s rights to effective education in the courts. We have outlined some examples of what professionals and academics can do to help, such as writing targeted articles or books, networking internationally, and conducting high quality research. All of these examples come from the desire to achieve open science, an approach to scientific evidence that is collaborative, honest, transparent, and rigorous (Dillenburger & Keenan, 2023).

There is another fundamental take-home message, though. Providing scientific evidence on its own is no guarantee that policy makers will embrace scientific findings in their decision making (Unumb, 2013; Virués-Ortega & Yu, 2018). This is particularly so in places where misrepresentation of ABA is endemic. Even if advocates manage to make some headway, they need to remember that those who take advantage of progress may not espouse the same humanistic principles that guided the founding fathers and mothers of applied behavior analysis (Morris et al., 2005).

Complications arise when procedures that are based in ABA are branded, sold, and promoted ahead of the science from which they stem (Freeman, 2003; Keenan et al., 2010). The warnings advanced then have resurged again in response to the consequences of commercialization within service provision (Garner et al., 2022). Furthermore, statements from the National Autistic Society (2023) as well the British Institute for Learning Disabilities ([BILD] 2022) show the erosion in confidence in ABA-based interventions that has been created by professionals in the UK wedded to one of ABA’s offshoots (Positive Behavior Support; PBS) and who appear to be distancing themselves from the knowledge-base in which these offshoots are grounded (see also PBS Academy, 2015). This is an extremely disconcerting turn of events given the statement by Horner (2021) in personal communication to a student of behavior analysis: “PBS is grounded in ABA, and from my view you cannot claim to be doing PBS without full engagement with the principles and practices associated with behavior analysis.” What is more, in his *Letters to a Lawyer*, Baer’s (2004) simple statement highlights the damage that is being done to the legacy of ABA by the unrelenting failure of professionals to disentangle bad practice from an accurate description of the science: “Everything I have described as typical ABA programming does not evoke negative emotions” (p. 14).

In conclusion, there is no reason why the 2021 BPS autism guidelines could not be revised into a better, more inclusive document. The BPS could seize the opportunity to evidence that it has truly aligned itself with the principles of open science by engaging seriously with a different scientific tradition and encouraging dialogue with recognised professional bodies in behavior analysis (ABAI, 2023; APBA, 2017; BACB, 2023). The recent Professional Standards Authority (PSA) accreditation of the UK-SBA register of behavior analysts (PSA, 2023) has now been added to that list (UK-SBA, 2023).The outcome of this dialogue would influence the development of social policies benefiting the public and would be consistent also with the values and work of both psychologists and behavior analysts. If this does not happen, then the aspirations for open science will remain merely that, aspirational. In the words of the Center for Open Science:

Science will not meet its potential until the research culture enables and supports contributors from all backgrounds and circumstances and contributions of all kinds based on the interests, skills, and resources available. Failure to achieve diversity and inclusion of all stakeholders in science will slow progress in discovery and translation of knowledge to solving humanity’s most pressing problems. (Centre for Open Science, 2022)

## Footnotes

1 The use of terms with regards to autism is important. Research has shown that “autism” or “on the autism spectrum” were the most acceptable terms, both for persons on the autism spectrum as well as professionals working with this population (Bury et al., 2020; Kenny et al., 2016; Singer et al., 2022; Taboas et al., 2022). Therefore, we are using identity- and person-focused terms interchangably.

2 The term "socially important” indicates that the individual target behavior should be aimed at enhancing the quality of life for the learner rather than requiring the learner to fit into controversial societal norms or expectations (Conine et al., 2022; Independent Forensic Expert Group [IFEG], 2020).

3 The abbreviation “ABA” used in the 2021 BPS guidelines is not spelled out. As a result, the uninitiated reader is left guessing what all the fuss is about.
